# Trust in the U.S. Government and Its Health Agencies in the Time of COVID-19

**DOI:** 10.3390/epidemiologia3020012

**Published:** 2022-03-22

**Authors:** Maraika Geisterfer-Black, Taylor Niemi, Leonie Neier, Victor G. Rodwin

**Affiliations:** 1 Global Studies Institute, University of Geneva, Rue des Vieux-Grenadiers 10, 1205 Geneva, Switzerland; taylor.niemi@etu.unige.ch; 2 Faculty of Economics and Behavioral Sciences, University of Freiburg, Friedrichstraße 39, 79098 Freiburg, Germany; leonie.neier@students.uni-freiburg.de; 3 Wagner School of Public Service, New York University, 295 Lafayette Street, New York, NY 10012, USA; victor.rodwin@nyu.edu

**Keywords:** COVID-19, institutions, trust, culture, politics, communication, public health, United States

## Abstract

This article examines the factors affecting Americans’ trust in their federal government and its health agencies during the COVID-19 public health crisis. More specifically, we examine the evolution of Americans’ trust in their government and health system and how, in the context of the COVID-19 pandemic response, it has been affected by multiple factors. Several academic journals, government policy recommendations and public health polls were evaluated to understand the public’s trust in the federal government and its health institutions. Public trust in institutions during a global pandemic is essential in influencing adherence to a pandemic response (both non-pharmaceutical and medical interventions). Americans’ trust in institutions is built and maintained by a variety of factors. We focus on: political polarization and involvement, media influence and health communications, history of systemic racism and socioeconomic inequalities, and pandemic fatigue. Based on the interplay of these factors, we conclude with recommendations for future pandemic response strategies.

## 1. Introduction

In March 2020, the lives of Americans, as they knew it, came to a grinding halt. The coronavirus pandemic, which had seemed, but a month earlier, to be a distant problem, tore through the world at an alarming pace. Unspared, the United States declared the coronavirus to be a national emergency on 13 March [1] and instigated widely implemented stay-at-home orders lasting, in some states, for months. Over the course of the last 20 months, more than 51 million people have been infected in the United States while 807,397 lives have been lost to the virus [1]. Today, the United States continues to be the global leader of confirmed COVID-19 cases and deaths, with both sitting at just shy of one-fifth of the world’s total numbers. The United States is one of the richest countries in the world, and yet the pandemic spiraled so quickly and devastatingly out of control within its borders.

On 7 January 2020, the Center for Disease Control and Prevention established their 2019-nCov Incident Management Structure to guide their response. This report “highlighted the need for a multidisciplinary public health approach—with surveillance, laboratory, and healthcare systems/networks, among others, intersecting and coordinating as part of a larger public health emergency response system” [1]. By 21 January, the CDC transitioned from incident management to agency-wide structure and activated their emergency response system. This is the highest level (level 1) response which includes multifaceted strategies to address the situation. These strategies include, but are not limited to, clinical mitigation, contact tracing, community mitigation, and surveillance [2]. For the purposes of this paper, the strategies focused on will be those that require active public participation. In the early months of the pandemic, it was largely unknown how lethal the virus would be and what best practices to use to curb infection rates while also accounting for the risks of social and economic upheaval [3]. Without a known cure or reliable treatment options, countries were left with the prospect of implementing non-pharmaceutical interventions that would greatly upend the lives of their citizens. In the United States, government restrictions ranged from widespread quarantine orders to reduced capacity in public places to mask-wearing indoors.

**Figure A1:**
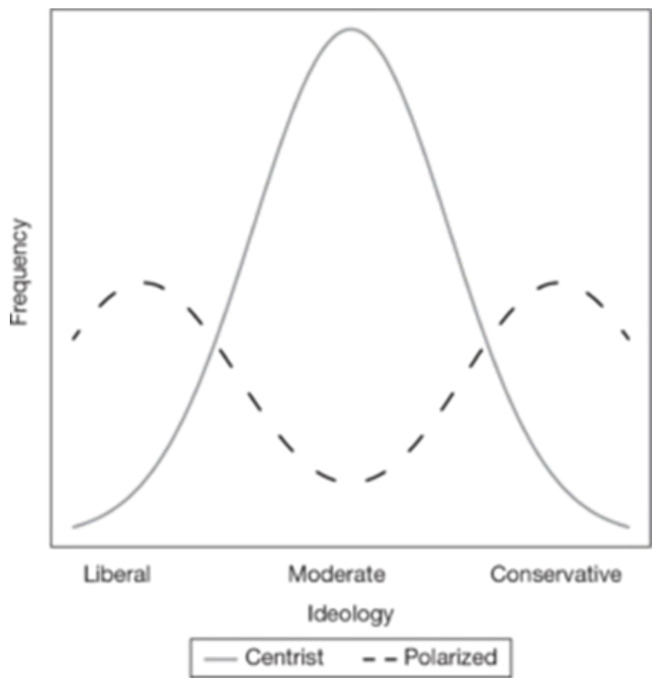
Distribution of centrist versus polarized politics.

For the first time, federal agencies had to contend with executing a scientific public health response alongside the responsibility of uniting the public in an emergency response plan that would present many social, political, and financial challenges. “Previous epidemics have shown that population compliance with government restrictions can make or break outbreak containment efforts. We also know that one of the major determinants in how well citizens comply with government recommendations is trust” [4] (p. 23). Public adherence to government-mandated and recommended measures quickly became a crucial aspect to managing the unfolding COVID-19 crisis. Institutional trust is defined as “the extent to which individuals accept and perceive institutions as benevolent, competent, reliable, and responsible toward citizens” [5] (p. 3) and identified as a significant predictor of public adherence [6]. Therefore, identifying barriers to establishing and maintaining institutional trust is paramount in understanding how to prepare a comprehensive public health strategy. According to David W. Baker, trust in medical systems and healthcare clinicians rests upon the assertion that, in healthcare contexts, the interests of patients are put above all else, including financial interests. Baker found that, “in a 1966 survey of adults in the US, 73% said they had great confidence in the leaders of the medical profession; but in a 2012 survey, only 34% said this” [7] (p. 2373). Additionally, a 2015 poll, “The Public’s Perspective on the United States Health System” conducted by the Harvard T.H. Chan School of Public Health, asked respondents to rate the level of trust they have towards recommendations put forth by different public health groups [8]. Respondents were asked if they had a great deal of trust, somewhat, or not at all. The Center for Disease Control and Prevention has the highest approval rating among health institutions with 52% of respondents reporting a great deal of trust in their health recommendations. Other health and government agencies lag with approval ratings dipping down to 33% [8] (Figure A1).

Evaluating institutional trust and understanding the factors that involve building (and maintaining) it is paramount during a global pandemic. Polls that exist today often show trust as a “this or that” concept; however, in reality, it is much more complex. Institutional trust can be gained or undermined in the wake of a global emergency and must be treated as a multifaceted issue, consisting of various factors that influence an individual’s evaluation of trust. This article examines the relationship between citizens and state within the context of managing a public health crisis. More specifically, this paper investigates, through the lens of the COVID-19 pandemic response, how relationships of trust are evaluated between government agencies and people in the United States. In the wake of the COVID-19 pandemic, there has been documented growing partisan differences in the strength of trust in public health officials. In most polls and reports published examining public trust, the most cited difference is across political affiliations [9]. Though political polarization and involvement is an important aspect to understanding the unique cultural context in which trust is evaluated in the United States, other factors also play an important role. Media influence and health communications play a crucial role in community building and perceptions of competence and reliability of public servants. A history of systemic racism and socioeconomic inequality resulted in disproportionate levels of distrust amongst marginalized members of society. Lastly, after months of social and economic upheaval, pandemic fatigue increased protests and public backlash.

Throughout the course of the COVID-19 pandemic, the United States response lacked coordination and cohesiveness in many areas. Looking specifically at the way strategies requiring public participation were implemented, institutional trust exists as a major barrier to successful management. Investigating the unique socio-cultural context within which institutional trust is constructed in the United States can help create a better public health plan for future national emergencies. Moreover, it could aid in generalizing the importance of cultural understanding for pandemic response frameworks in other nations as well.

## 2. Materials and Methods

Examining the United States as a case study, a scoping review of peer-reviewed and grey literature was conducted to determine and explain the challenges that state and public health institutions faced in establishing strong public health participation. With the goal of providing a comprehensive understanding of these challenges, interdisciplinary literature was sourced from public health, political science, psychology, sociology, and healthcare. Using a structured search strategy, literature was retrieved from academic databases, Google Scholars (https://scholar.google.com, accessed on 6 October 2021) and JSTOR (https://www.jstor.org, accessed on 16 October 2021), and the Web for grey literature. An initial search on the databases was conducted with an inclusion criterion of keywords that needed to be present in the title (COVID-19; Trust; United States). Following this stage, the scope was broadened to include articles focusing on public participation in public health measures. Due to the limited amount of COVID-19 specific literature, a third search was conducted to include studies done in other countries. Literature that focused on vaccine adoption and country-specific factors not common to the United States were excluded.

The investigations revealed quantitative data in the form of polls and surveys which were used to develop an accurate picture of the state of institutional trust that exists in the United States today. The qualitative studies were analyzed to understand the components of institutional trust and adherence to public health measures necessary for COVID-19 mitigation. Public health policies published by the United States (CDC) were used to ascertain the widespread obligatory non-pharmaceutical interventions in the United States and their varied usage across states. The pandemic strategy pages of the CDC website were additionally analyzed to identify inclusion criteria in the strategy (namely whether trust in public systems was addressed as a barrier). All the findings from the literature were then assembled and analyzed to provide a holistic understanding of the barriers public health institutions and the government faced in establishing and maintaining trust with the public.

## 3. Results

### 3.1. Political Polarization and Involvement

**Figure A2:**
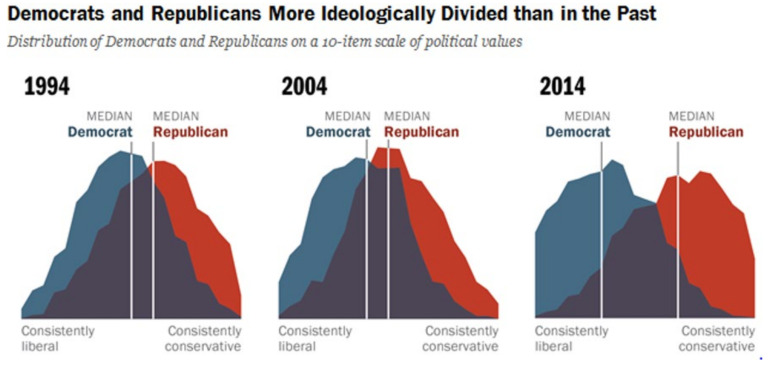
Polarization figures 1994–2014. Ideological consistency was established with a scale of 10 political value questions. The blue and red area represent the ideological distribution of Democrats and Republicans respectively. The purple area represents the overlap between the two. Republicans and Democrats include Republican- or Democratic-leaning independents respectively [11].

**Figure A3:**
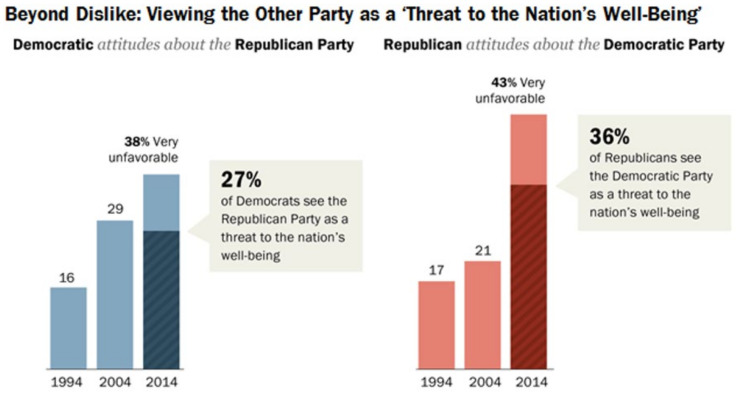
Views of opposing parties 1994–2014. The questions for this study were asked only in 2014. Republicans and Democrats include Republican- or Democratic-leaning independents respectively [11].

Political polarization is the process by which opposing views become more common and severe over time. Nolan McCarty, in his book Polarization: What Everyone Needs to Know, writes that in the United States, it is assumed that political ideologies are situated along a continuum between liberal and conservative, creating ranges between left and right [10] (p. 10). The distribution of political orientation in a region that is not polarized will more likely resemble a normal distribution while politically polarized regions will show more bimodal distributions, as shown in Figure A1. In the United States, political polarization is characterized by increasing numbers of voters describing themselves as consistently aligned with one party or another, more negative opinions towards those belonging to opposing parties, and a stronger desire to associate only with same-party voters. According to the Pew Research Center, “Today, 92% of Republicans are to the right of the median Democrat, and 94% of Democrats are to the left of the median Republican. In each party, the share with a highly negative view of the opposing party has more than doubled since 1994” [11] (lines 9–16). Figure A2 and Figure A3 illustrate the growing partisan divide and animosity, respectively.

Political polarization unites individuals and groups sharing the same values while simultaneously dividing populations along ideological lines. “Nearly two-thirds (63%) of consistent conservatives and about half (49%) of consistent liberals say most of their close friends share their political views. People on the left and right are also more likely to say it is important to them to live in a place where most people share their political views” [11] (lines 96–104). This process of selecting and curating social networks that confirm and reinforce beliefs contributes to growing identity polarization in line with political affiliation. Roderik Rekker, in the context of political polarization over science, describes two interrelated processes that weaken belief in scientific claims. “Through psychological science rejection, people can implicitly disregard scientific facts that are inconsistent with their political identity. Alternatively, citizens can engage in ideological science rejection by adhering to a political ideology that explicitly contests science” [12] (p. 352). These two processes can help to shed light on growing partisan divides in issues such as belief in climate change or vaccine uptake during the pandemic. For the latter, according to a recent Gallup poll, “40% of Republicans ‘don’t plan’ to get vaccinated versus just 3% of Democrats” [13] (lines 27–28). The danger of identity polarization is the increased strength of identities constructed based on opposition to beliefs held by members of opposing identities (political parties).

Throughout the pandemic, these trends have undermined public health efforts by creating ideological sides debating the existence of the virus itself, the necessity for non-pharmaceutical interventions, and the moral implications of economic versus human costs. These debates eroded productive democratic decision-making by taking away all common ground. “Democratic debate over policy requires at least some basic agreement on facts [which] are the ‘currency of citizenship’ that prevent debates from becoming disconnected from the material conditions they attempt to address” [12] (p. 353). During the pandemic, in the United States, enough people were strongly focused on disregarding those basic facts which ultimately resulted in uncoordinated implementation of measures and fractured public adherence to those measures put in place.

In addition to the general trend of political polarization in the United States, the COVID-19 pandemic response was strongly affected by political involvement both at the federal and state level. Management efforts on behalf of public health agencies were often disrupted by misleading and sometimes false information provided by political leaders. For example, multiple misinformation themes centered around the ‘miracle cures’ for coronavirus, most notably the advocacy for the use of hydroxychloroquine as a treatment despite lack of support by physicians [14]. “Despite the important actions taken by federal public health agencies to curb the pandemic, public trust in these agencies such as the Centers for Disease Control and Prevention, the National Institutes of Health, and the Food and Drug Administration, and their world class career scientists has decreased during the pandemic” [15] (lines 2–6). Mitigating a public health crisis such as the coronavirus pandemic requires participation by the public, which remains contingent upon the trust the public holds in federal agencies. The above-mentioned agencies struggled with the constantly changing narrative of the Trump administration. Some notable examples include: (1) Thursday, 27 February, Trump claimed the outbreak would only be temporary while Anthony Fauci (director of the National Institute of Allergy and Infectious Diseases) warned of higher rates of community-spread infections; (2) Saturday, 4 July, Trump announced that “99% of COVID-19 cases are totally harmless” while the WHO confirmed 15% of cases to be severe and 5% to be fatal; (3) On several occasions, Trump intimated that the media were exaggerating the impacts of COVID-19 leading up to elections when cases were nearing an all-time high at the end of October 2020, reaching almost 100,000 daily cases; and (4) Trump cited a CDC article and declared they found that 85% of mask-wearers caught the virus. The CDC study in question did not attempt to estimate the likelihood of infection in mask wearers but found that people who tested positive were more likely to have gone out into the community and, of those, 85% wore a mask always or often [16] (whole text, see: Table A1 in Appendix A). This does not represent an exhaustive list of the times the Trump administration directly contradicted scientific evidence or recommendations put forth by American federal public health agencies. These examples demonstrate the magnitude of political involvement in the COVID-19 response and served to make the pandemic a political playing field rather than the national crisis that it was.

### 3.2. Media Influence and Health Communication

In a world increasingly experienced online, social media plays an instrumental role in understanding and guiding social dynamics. Social media so often acts as an agent of knowledge, cultural beliefs, and self-expression. The ability to curate experiences, identities, and truths in the way that is possible today is completely unprecedented. Not only is that an option for people in power, people in academia, and others who typically share the public ear, information can be widely disseminated by anyone with a laptop and an internet connection. Both for better and for worse, throughout the pandemic, “because of strict physical distancing measures, people are heavily reliant on maintaining connectivity using global digital social networks, such as Facebook or Twitter, to facilitate human interaction and information sharing about the virus” [17] (p. 277). Building on the knowledge gained in the previous section, media organizations are not immune to the effects of political polarization with some media sources openly leaning to the left or to the right. In Dhanani and Franz’s exploration into the role of news consumption and trust in public health leadership, they found that some American news sources provided science-backed information about COVID-19 while others engaged in tactics such as spreading misinformation, scapegoating, and minimizing the pandemic’s effects. “These differing messages have not only created deep fissures among the public but have also resulted in harmful behaviors such as refusing to comply with recommended practices to stem transmission, [and] violence towards those who try to enforce such practices” [9] (p. 2). This type of mixed messaging in mass media in conjunction with the ideological network selection on social media reinforces value and belief boundaries that create impenetrable echo-chambers [9] (lines 90–92).

Inconsistent messaging in mass and social media also gives rise to the phenomenon of ‘alternative facts’, something that is seen more and more frequently. “The idea of legitimacy has changed in the context of social media platforms. Users increasingly see trusted individuals within their peer networks who support production and exchange of valued information as authoritative sources of information” [17] (p. 277). The onus of perceived legitimacy rests less and less on trusted, objective scientific research and more on authoritative gravitas. Furthermore, when scientists engage in health communication, the general philosophy is one of caution. Scientific communication is committed to not overstating confidence in findings, openly disclosing limitations to results, and revising information when proven incorrect. By contrast, those who peddle pseudoscientific concepts or encourage conspiracy theories are steadfastly confident in their beliefs and never back down, even when presented with proof that contradicts their opinions [18]. This helps to create the illusion that truth is subjective and all opinions are valid. An example of this is the advent of direct-to-consumer drug advertisements. Parmet and Paul explain that advertising medications directly to consumers unintentionally overrides the sense of reliability in the clinical expertise of medical professionals. Grounded in ideals of informed consent, “patients have come to believe that their views ‘count’ as much as those of their health provider” [19] (lines 43–53). This can be additionally seen in the rise in hesitancy surrounding the COVID-19 vaccine. Calls to “do your own research” are common on social media platforms where “truth feels fleeting; claims are more or less persuasive based not on the accumulation of rigorous science or the credibility and expertise of the speaker but on the celebrity, political party, or intuition of speaker and listener” [19] (lines 34–36). Mounting a coherent defense against a highly contagious virus in an informational atmosphere depleted of credibility and reliability makes it impossible to create strong institutional trust.

The media influences the way people think, the way people construct identities and beliefs, and the way people come to understand knowledge and engage with knowledge bearers. The media has created an environment where everyone is free to be heard. Yet, with that comes the opposite opportunity of being free to be ignored. Instead of being plagued by a lack of information, people are now inundated with far too much to make sense of it all. Competing narratives about scientific data, false information, consumer-driven policies, and social media algorithms that create ideological echo chambers all come together to undermine the value of scientific facts and the institutions that stand by them.

### 3.3. Systemic Racism and Socioeconomic Inequality

Health equity is essential to the overall health and wellbeing of a nation. When a group is disproportionately affected by their health system, it creates lack of trust and efficacy in public health campaigns. In the United States, there is a lower level of trust amongst minority communities, especially black and indigenous groups who have also experienced the highest death tolls in the US [20] (line 11). Historically, these communities have been marginalized and used to their own detriment by the United States’ public health system. One of the most notable examples of this was the Tuskegee Syphilis Study conducted by the United States Public Health Service in 1932. This study followed 400 African American men infected with syphilis in Tuskegee, Alabama. “The subjects received heavy metals therapy, standard treatment in 1932, but were denied antibiotic treatment therapy when it became clear in the 1940s that penicillin was a safe and effective treatment” [21] (lines 12–15). The systemic racism present in the United States allowed for African Americans and other minority groups to be inhumanely treated within the public health system to advance scientific research, among other reasons. Consequently, the baseline level of trust for minorities entering the pandemic was already far behind that of white Americans.

The COVID-19 pandemic has brought social and racial injustices as well as health inequalities to the center stage as it becomes increasingly hard to ignore the additional barriers to healthcare and health disparities that minorities face. “African Americans are up to three times more likely to contract COVID-19, and up to six times more likely to die from a COVID-19 infection compared with non-Hispanic whites in the U.S.” [22] (pp. 1–2). These numbers illustrate the failure of public health institutions to protect the increased vulnerabilities of certain communities. Furthermore, discrimination towards Americans of East Asian descent increased a great deal throughout the pandemic. The Washington State Journal reported that, “President Trump deliberately calling the novel virus a name that has been frowned upon by critics, who say its usage could lead to increased discrimination and racism toward Asian Americans—a marginalized group with a long history of being scapegoated amid public health crises” [23] (lines 6–9). This dangerous rhetoric has led many Asian Americans to report situations of physical and verbal abuse. With the president fueling ongoing racist language, Asian Americans are finding themselves in a society which rejects and blames them. This perception, joined with a historical precedent of marginalized groups being blamed for spreading illnesses and diseases, has led to repeated discrimination amongst minority groups [23] (lines 42–44). The effect of this public discrimination and blaming has deleterious effects on the level of trust that these marginalized groups have in the public system [22,23].

For minorities living in the United States, being confronted with a public health crisis such as the pandemic can bring up traumatic memories of engaging with the healthcare system (individual level) and reinforce intergenerational trauma (community level). “Contemporary research shows that socioenvironmental discrimination and recent healthcare experiences with medical professionals influence medical mistrust among African American men. Experimental research […] reveals that exposure to racial discrimination significantly increases medical mistrust among African Americans” [24] (p. 23). Racial and ethnic minorities contended with increased barriers to healthcare access which, compounded with a history of systemic abuse and current experiences of increased discrimination, pushed them further away from being able to trust the system that was supposed to have their best interests at heart.

At the intersection of race and inequality stands, inexorably, socioeconomic status. “Research has shown that race and ethnicity in terms of stratification often determine a person’s socioeconomic status” [25]. Though not all minorities are living in low socioeconomic conditions, a correlation exists between the two, and understanding healthcare inequalities requires an acknowledgment of socioeconomic status. The COVID-19 pandemic created another barrier to access of care, particularly for those who lost their health coverage due to unemployment. Considering this system, “people of lower SES are more likely to have worse self-reported health, lower life expectancy, and suffer from more chronic conditions. They also receive fewer diagnostic tests and medications for many chronic diseases and have limited access to health care due to cost and coverage” [26] (p. 169). In the context of the pandemic, the impacts of lockdowns and business closures were felt more strongly among low socioeconomic groups. People with higher education are more likely to have in-office jobs which, throughout stay-home orders, are more easily converted to remote work. Manual laborers and others engaged in non-essential work that requires in-person service were not afforded that same luxury [27]. The unpredictability of work for low-wage workers during the COVID-19 pandemic further perpetuates inequality within the workforce and accelerates restricted opportunities which impacts marginalized groups [27].

### 3.4. Pandemic Fatigue

Pandemic fatigue is defined by the WHO as, “a demotivation to follow recommended protective behaviors, emerging gradually over time and affected by a number of emotions, experiences and perceptions” [28]. Pandemic fatigue is not surprising, given the severity of the pandemic and the imposition on individual’s daily lives. While the American response to the pandemic has taken different paths over the course of time, shared is a sentiment of weariness and frustration regarding the length of restrictions. Petherick et. al., “observed a clear and robust association between interpersonal trust and change over time in adherence to physical distancing as the months pass, to sustain their own compliance. It appears that people need to trust that strangers will also physically distance” [29] (p. 1156). Long gone are the times of unity which drove Americans to stay inside, slow the spread and protect community health. With pandemic fatigue, a deep desire to return to some sense of normalcy drives individuals to return to restaurants, travel, and interact with each other once again.

There are serious consequences to this shared pandemic fatigue experience. As exhaustion and impatience for a “return to normal life”, individuals begin disobeying health recommendations and COVID-19 cases begin to soar. Slowly, fear of the virus turns into frustration, and behaviors begin to change. Possible reasons for this decline in adherence include: the economic burden these behaviours cause for the individual, adjustments to risk assessments, and varying intensity of enforcement measures [29] (p. 1155). These three factors possibly contribute to behavioural changes associated with pandemic fatigue. It then becomes increasingly difficult for health officials to manage a pandemic and curb rising cases when individuals are not abiding by the protective measures set in place. ”Life and literature have taught us that desirable but costly healthy behaviours are easier to initiate than to sustain. It is plausible to suspect that physical distancing is not only psychologically demanding but also cost accumulating” [29] (p. 1155–1156). A worldwide analysis of non-pharmaceutical measures found that adherence to low-cost measures such as mask wearing increased throughout the pandemic; however, high-cost measures such as social distancing decreased [29] (p. 1155). High-cost measures were characterized as having significant social and economic consequences. Bodas and Peleg concluded that “the failure of decision makers to appropriately address the economic constraints imposed on the public during prolonged disasters, such as COVID-19 outbreak, is likely to lead to a reduction in public trust in the government and to a decrease in societal resilience” [30] (lines 11–14). Pandemic fatigue is still a relatively new concept and challenge within the context of COVID-19; however, it presents a unique struggle to public health officials attempting to convey the continued seriousness of the pandemic.

## 4. Discussion

The United States experienced the COVID-19 pandemic through a political lens. No aspect of the pandemic was devoid of politics, and the government response was influenced as heavily, if not more, by the factors listed above. The government handling of the pandemic was heavily impacted by the feedback loops between the political polarization, media influence, systemic barriers, and pandemic fatigue. Trends at the state and federal levels demonstrated political loyalty overriding public health concerns as authorities aligned their response strategies with their political affiliation rather than proven public health measures. The fractured response by the states undermined the authority of scientifically backed health measures by calling into question their reliability and validity. “The severity of this crisis is particularly sensitive to public opinion, given that behavioural change at the individual level is integral to successfully slowing the spread of the virus” [31] (p. 1). Due to the high levels of political polarization, it is unlikely that individuals will change their attitudes towards public health measures unless political consensus deems it necessary [32] (pp. 147–174). The inconsistency in strategy and messaging was irresponsible in terms of public health practice, and further divided the population along political-ideological lines. The next few years will be characterized by these divisions, and the next administrations will be defined by their actions towards reconciliation or further severing relationships.

The COVID-19 pandemic is the first global pandemic seen in over 100 years. Hopefully, it will be the last for this century; however, with increasing globalization, the risks of further pandemics are too many to be ignored. Identifying weak spots in a country’s health and pandemic response strategy is critical for building stronger health systems and achieving better health outcomes. Continuous long-term distrust in health institutions will create ever more challenging landscapes when it comes to pandemic responses and general health recommendations. This article provided a brief overview of the four core areas that American public health institutions need to target to improve their levels of public trust. This raises new questions such as: does everyone experience pandemic fatigue equally? What are, if any, the protective factors against institutional distrust for marginalized communities? What are the specific risk factors for rejecting scientific information and health communications? Further research is required to gain a deeper understanding of each area on its own, as well as a more nuanced perspective of how each area interacts and affects the others. Once more targeted research is achieved, only then can effective policies be put into place. Some preliminary research-focused recommendations are suggested below.

Recommendations:

1. Polls/surveys that investigate public health trust should be context specific, direct, and organized with as minimal bias as possible.
2. CDC (or national equivalent) should address public adherence barriers and challenges with specific strategies to respond to them.
3. Continuous efforts in reducing (researching and understanding) racial and socioeconomic inequalities to create a more equitable, healthcare system.

Polls and surveys that investigate public health trust should be context specific. This could include sections within the poll that attempt to identify which barriers the individuals submitting the poll face (if any). Questions should consider each dimensional context mentioned above. These polls should be conducted regularly, and the results should drive research with solution-based actions. The data from these polls should be used to inform public health plans by the CDC (or national equivalent), which should have a section in the pandemic response plan to include managing public perception of health institutions. “Information campaigns directed at, and designed for, all segments of the US population should be introduced and sustained to reduce vaccine hesitancy, and additional resources should be provided to the CDC to counter misinformation and conspiracy theories promulgated on social media and by the press” [33] (p. 210) Integrating a cultural context to government institutional response plans will positively impact action regarding public health trust. Lastly, there exists within the United States a fragmented health system which is convoluted, selective, and expensive [34]. There should be continuous efforts in researching, understanding, and reducing inequalities that are evident in today’s system. “Enhancing resilience to future disease outbreaks requires longer-term work to create high-quality healthcare systems and build community trust. Health systems need appropriate financing, not only to prepare for future pandemics but also to ensure that at all times, all people have access to the health services they need” [35] (p. 978) Daszak, et al., propose strengthening “access to healthcare by expanding the number of people with a social safety net of health insurance coverage as a first step in dealing with current inequities in access” [33] (p. 209) The American cultural identity of “all for one, and one for all” should be applicable to the system of health on which the nation relies. An improved healthcare system that does not discriminate and is equitable will provide a strong foundation for a healthier population, aid in future pandemic management, and reduce socioeconomic health inequalities.

## 5. Conclusions

As discussed in this paper, non-pharmaceutical interventions such as stay-at-home orders, mask wearing, and social distancing are highly effective methods for curbing community transmission. The success of those methods is contingent upon public adherence to measures, and public adherence itself depends largely on how trustworthy health authorities are perceived to be. In the United States, the state of public trust in government and health authorities is unstable and insecure. In the case of the COVID-19 pandemic, public health management efforts encountered political polarization, media influence on health communications, systemic racism and socioeconomic inequality, and pandemic fatigue as barriers to implementing a coordinated response and garnering public support for that response. The United States’ pandemic response cost the nation not only millions of dollars but also hundreds of thousands of lives. This experience can, and should, be used as a learning opportunity for the government to begin addressing the serious inequalities that exist in the country today. Understanding the reasons behind institutional distrust should be a priority for American public health in order to strengthen pandemic responses in the future and improve the general state of healthcare for the population.

## Data Availability

Not applicable.

## Appendix A

**Table A1:** Examples of four presidential claims versus facts.

Presidential Claim	Fact
27 February 2020-Trump claimed the outbreak (coronavirus) would only be temporary	Anthony Fauci (director of the National Institute of Allergy and Infectious Diseases) warned the public of higher rates of community-spread infections
4 July 2020-Trump announced that “99% of COVID-19 cases are totally harmless”	WHO confirmed 15% of cases to be severe and 5% to be fatal
Trump stated that the media were exaggerating the impacts of COVID-19 leading up to elections	During election season (October 2020), cases reached an all-time high at a rate of nearly 100,000 new cases per day
Trump cited a CDC article and intentionally distorted its findings by declaring they found 85% of mask-wearers caught the virus	CDC cites mask wearing to be a highly efficient method of reducing transmission and preventing COVID-19
